# Polyvascular Disease and the Incidence of Cancer in Patients with Coronary Artery Disease

**DOI:** 10.31662/jmaj.2022-0098

**Published:** 2022-09-26

**Authors:** Makoto Suzuki, Hitonobu Tomoike, Zhehao Dai, Toru Hosoda, Tetsuya Sumiyoshi, Saichi Hosoda, Mitsuaki Isobe

**Affiliations:** 1 Department of Cardiology, Sakakibara Heart Institute, Tokyo, Japan; 2 Hoshinooka Cardiovascular Clinic, Ehime, Japan

**Keywords:** atherosclerosis, cancer, cardio-oncology, polyvascular disease, coronary artery disease, cohort study

## Abstract

**Introduction:**

Based on the possible relation of atherosclerotic cardiovascular disease to the development of cancer, we examined whether polyvascular disease, as a surrogate marker of the severity of atherosclerosis, is associated with the incidence of cancer in patients with coronary artery disease (CAD).

**Methods:**

A total of 8,856 patients with CAD between January 2009 and July 2014 were eligible for this observational study. Two cohorts were established based on the presence or absence of polyvascular disease (i.e., polyvascular disease and CAD only) and tracked for the incidence of cancer and all causes of death. Polyvascular disease was defined when accompanied by diagnosed aortic and/or peripheral arterial disease or other arterial diseases at enrollment.

**Results:**

With a median follow-up of 1,095 d, the incidence of cancer was markedly higher in the cohort of 716 patients with polyvascular disease than in the cohort of 8,140 patients with CAD only (8.8% vs. 4.9%, *P* = 0.0001). A large difference in the incidence of cancer was also found in accordance with a number of the coexisting vascular disease with CAD. With the adjustment of shared common risks, polyvascular disease was an independent contributor to the incidence of cancer (hazard ratio, 1.362; 95% confidence interval [CI], 1.029-1.774). In a total of 548 patients (6.2% of participants) died during follow-up, and all-cause, cardiovascular, and cancer mortalities were all higher in the cohort with polyvascular disease than in the cohort with CAD only.

**Conclusion:**

The presence of polyvascular disease may be associated with the incidence of cancer in patients with CAD, implying a pivotal role of the severity of atherosclerosis in cancer development (ClinicalTrials.gov. number: NCT04198896).

## Introduction

Healthcare epidemiology has been contemporarily validated to acquire sustainable health longevity in the prevention and treatment of atherosclerotic cardiovascular disease and cancer, which are the two leading causes of death among non-communicable diseases ^(1)^. Unhealthy lifestyle behaviors and their associated diseases, including obesity, tobacco smoking, hypertension, and diabetes mellitus, among others, may share many common risk factors for the development of both atherosclerotic cardiovascular disease and cancer, with the possibility of overlapping causes for both diseases ^(2), (3)^. Recent international perspectives further endorse the practical impact of various cardiovascular diseases on cancer development ^(4), (5)^. We also reported the possibility of an association between atherosclerotic cardiovascular disease itself and the incidence of cancer ^(6)^. Based on these findings, we then investigated how the severity of atherosclerosis may affect cancer development. The presence of polyvascular disease, namely, the coexistence of atherosclerotic diseases in two or more vascular trees, is now well-recognized as a high-risk phenotype of atherosclerosis for subsequent cardiovascular events ^(7), (8)^. Thus, the present study evaluated whether the presence of polyvascular disease, as a surrogate marker of the severity of atherosclerosis, is associated with the incidence of cancer in patients with coronary artery disease (CAD) using the Sakakibara Health Integrative Profile (SHIP) surveillance system.

## Materials and Methods

### Study patients

As previously reported ^(6)^, the SHIP surveillance system was launched in 2006 to annually track subsequent clinical incidents requiring hospitalization, including not only cardiovascular diseases but also non-cardiovascular diseases such as cancer, pneumonia, and fracture, with the purpose of improving healthy life expectancy in patients referred to the Sakakibara Heart Institute. Since 2009, medical records of invasive cardiovascular treatments such as catheter and/or surgical interventions in our institute were also tracked. In the present study, a total of 9,092 patients with CAD who were identified from the SHIP surveillance system between January 2009 and July 2014 were extracted and followed-up by the end of October, 2019.

### Data collection and design

As previously reported ^(6)^, CAD includes acute coronary syndrome, previous myocardial infarction, and stable ischemic heart disease. Acute coronary syndrome was diagnosed according to the urgent algorithm composed of symptomatic abnormal ST segments in the 12-lead electrocardiogram, with abnormal cardiac enzyme spillover, and an urgent coronary angiogram. A previous myocardial infarction and stable ischemic heart disease were diagnosed by invasive and non-invasive cardiac examinations for the assessment of myocardial ischemia and viability. The appropriate use of invasive coronary therapies (i.e., percutaneous coronary intervention and/or coronary bypass surgery) was decided by the heart team. In accordance with the previous studies ^(7), (8)^, polyvascular disease was defined as the presence of any diagnosed arterial diseases, including aortic disease, peripheral arterial disease, and other arterial diseases. A diagnosis of aortic disease, which included acute aortic syndrome and/or aortic aneurysm, was made by imaging modalities, mainly, computed tomography, or medical records, including invasive treatments such as arterial bypass graft and/or endovascular replacement. Peripheral arterial disease was diagnosed with the following findings: symptomatic lower limb ischemia, such as intermittent claudication with an ankle-brachial index less than 0.9 and with an abnormal vascular echocardiogram or medical records of any vascular treatment, including amputation. Other arterial diseases were any atherosclerotic cardiovascular disease, except aortic and/or peripheral arterial disease, including atherosclerotic renal artery stenosis, subclavian artery stenosis, and carotid artery disease, mainly diagnosed by measurements with duplex ultrasonography and/or computed tomography angiography according to the established guideline ^(9)^. Briefly, atherosclerotic renal artery stenosis was defined with a duplex Doppler peak systolic velocity above 200 cm/s or more than 70% stenosis at the renal ostial arteries, subclavian artery stenosis by a duplex of high systolic velocity, occasionally with a reversal flow in an ipsilateral vertebral artery, followed and an angiographic significant subclavian stenosis, and carotid artery stenosis by more than 50% stenosis based on the North American Symptomatic Carotid Endarterectomy Trial definition. If aortic and/or peripheral arterial disease were present with any other arterial diseases, they were diagnosed as aortic and/or peripheral arterial disease. Based on the assumption that the presence of polyvascular disease is a surrogate marker of the severity of atherosclerosis, the study patients were categorized into the two cohorts with and without polyvascular disease, namely, the cohort with polyvascular disease and with CAD only, at enrollment in the SHIP surveillance system to evaluate the association between the severity of atherosclerosis and cancer development. Furthermore, after dividing the cohort with polyvascular disease into the two subsets in accordance with the coexistence of one vascular disease (i.e., aortic disease, peripheral arterial disease, or other arterial diseases) or two vascular diseases (i.e., both aortic and peripheral arterial diseases), the incidence of cancer was also evaluated. The present study did not include cerebrovascular disease in the category of polyvascular disease because our institute did not treat cerebrovascular disease patients and, thereby, had practical difficulties classifying causes of cerebrovascular disease into ischemic (i.e., lacunar infarction, atheroma-embolic infarction, and cardio-embolic infarction) or hemorrhagic stroke. Demographic and clinical characteristics, including the presence of hypertension, dyslipidemia, diabetes mellitus, chronic kidney disease, and chronic obstructive pulmonary diseases, in accordance with the universal definitions ^(10), (11)^, were examined, and a history of cigarette smoking was also confirmed. Using the SHIP surveillance system, medical records of invasive therapies by the end of follow-up and medications at the final follow-up were extracted. All organ sites of cancer and causes of death during the follow-up periods were also identified. As one of the regulations for the managements of the SHIP surveillance system, wherein there is a lack of any contact to the patients as to their clinical status for two years, the tracking was automatically discontinued.

### Statistical analysis

Continuous variables are presented as means ± standard deviation or medians with interquartile range. Differences between the two cohorts with CAD only and polyvascular disease, or the two subsets in the cohort with polyvascular disease were compared using Student’s *t*-test or the Mann-Whitney U test for continuous variables and Fisher’s exact test for categorical variables. Differences in three or more corresponding variables were assessed by one-way analysis of variance for continuous variables and Pearson’s test for categorical variables. The cumulative probability of cancer development or all-cause death during follow-up was examined using Kaplan-Meier curves with the log-rank test and/or the Wilcoxon test for comparison between the two cohorts with CAD only and polyvascular disease. The incidence of cancer in the subsets of the cohort with polyvascular disease were also compared with the cohorts with CAD only using Kaplan-Meier curves with the log-rank test and/or the Wilcoxon test. To assess the association of polyvascular disease with the incidence of cancer, adjustment for differences in clinical characteristics was performed using propensity score matching, with measurement of the C-statistic to evaluate the ability to control for confounding bias with a range of 0 to 1, with a higher value indicating well-controlled. A Cox proportional hazards regression analysis was carried out to assess the association of multiple risk co-factors with the incidence of cancer and also to test for the interactions of different clinical characteristics between the two cohorts with CAD only and polyvascular disease for the presence of polyvascular disease on the incidence of cancer. A two-tailed *P*-value of <0.05 was considered to indicate a significant difference. Statistical analyses were performed using JMP version 11.2.1. (SAS Institute Japan Inc. Tokyo, Japan). The SHIP surveillance system complied with the Declaration of Helsinki and was approved by the local ethics committee on September 16^th^, 2003, in the Sakakibara Heart Institute (no. 11000304) ^(6)^. All patients gave written, informed consent to be included in this surveillance system.

## Results

### Characteristics of the study patients

**Figure 1.**
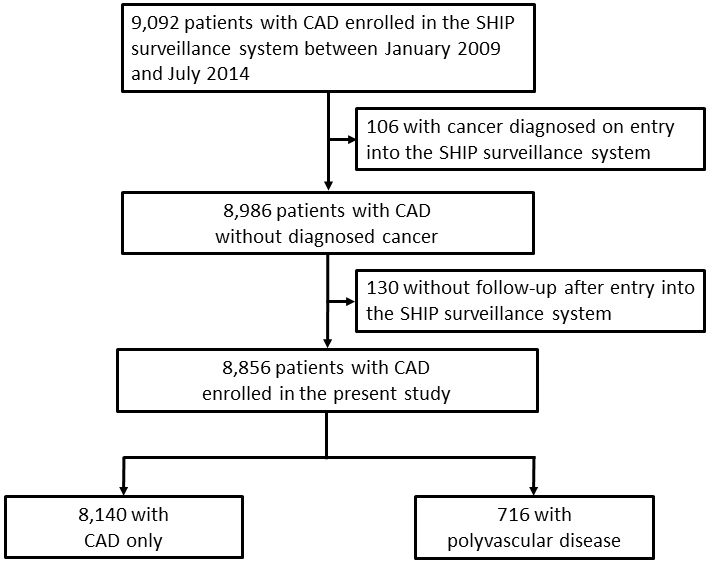
Flow diagram of the study patients. CAD, coronary artery disease; SHIP, Sakakibara Health Integrative Profile.

Of the 9,092 patients (74% male, age 70 ± 12 years) with CAD, a total of 8,856 patients were eligible for the present study because 106 patients had cancers on enrollment in the SHIP surveillance system, and 130 patients have not had any contact to us after enrollment. A median follow-up was 1,095 d with a maximum of 3,527 d. These patients were categorized into the two cohorts, 8,140 with CAD only and 716 with polyvascular disease, to compare the incidence of cancer (Figure 1). Table 1 shows the demographic and clinical characteristics of the patients at enrollment, records of invasive coronary therapies by the end of follow-up, and final medications on both crude and propensity score-matched analyses. In the crude analysis, demographic characteristics showed more elderly patients, more male patients, and high prevalence of hypertension, chronic kidney disease, chronic obstructive pulmonary disease, previous stroke, and cigarette smokers in the cohort with polyvascular disease than that in the cohort with CAD only. As to the medical history, previous myocardial infarction and heart failure were observed more frequently in the cohort with polyvascular disease than that in the cohort with CAD only. During the follow-up periods, 4,514 patients, 51% of the study patients, underwent percutaneous coronary intervention and/or coronary artery bypass grafting for the treatment of CAD, and their proportions were not different between the two cohorts with CAD only and polyvascular disease (51% vs. 54%, *P* = 0.0723), except for a higher percentage of coronary bypass grafting in the cohort with polyvascular disease than that in the cohort with CAD only. Of those treated with invasive coronary therapies, 308 of 4,126 patients in the cohort with CAD only and 32 of 388 patients in the cohort with polyvascular disease underwent both percutaneous coronary intervention and coronary artery bypass grafting (7% vs. 8%, *P* = 0.5469). Aortic disease was the main coexisting vascular disease in the cohort with polyvascular disease, and peripheral arterial disease was next. As for the final medications, differences in prescriptions of anticoagulants, diuretics, and statins were observed between the two cohorts (Table 1).

**Table 1.** Clinical Characteristics of the Two Cohorts, with Coronary Artery Disease (CAD) Only and with a Polyvascular Disease.

Characteristic	Overall	Crude analysis		*P*-value	Propensity score-matched analysis		*P*-value
		CAD only	Polyvascular disease		CAD only	Polyvascular disease	
	N = 8856	N = 8140	N = 716		N = 713	N = 713	
Age, y	70 ± 11 (71, 63-78)	69 ± 12 (71, 62-77)	74 ± 9 (75, 69-80)	<0.0001	74 ± 9 (75, 69-80)	74 ± 9 (75, 69-80)	0.9416
Male, no. (%)	6659 (74.1)	5980 (73.5)	579 (80.9)	<0.0001	577 (80.9)	576 (80.8)	0.9463
Body mass index, kg/m^2^	24 ± 3 (24, 22-26)	24 ± 3 (24, 22-26)	24 ± 3 (24, 22-26)	0.5601	24 ± 9 (24, 22-26)	25 ± 8 (24, 22-26)	0.8306
Hypertension, no. (%)	4915 (55.5)	4444 (54.6)	471 (65.8)	<0.0001	475 (66.6)	468 (65.6)	0.6953
Dyslipidemia, no. (%)	5021 (56.7)	4637 (57.0)	384 (53.6)	0.0907	434 (60.9)	383 (53.7)	0.0063
Diabetes mellitus, no. (%)	2384 (26.9)	2174 (26.7)	210 (29.3)	0.1351	220 (30.9)	209 (29.3)	0.5253
Chronic kidney disease, no. (%)	949 (10.7)	785 (9.6)	164 (22.9)	<0.0001	154 (21.6)	161 (22.6)	0.7018
Chronic obstructive pulmonary disease, no. (%)	159 (1.8)	127 (1.6)	32 (4.5)	<0.0001	29 (4.1)	29 (4.1)	1
Previous stroke, no. (%)	225 (2.5)	188 (2.3)	37 (5.2)	<0.0001	22 (3.1)	36 (5.1)	0.0805
Cigarette smoking, no. (%)	4300 (48.9)	3840 (47.2)	460 (64.3)	<0.0001	460 (64.5)	457 (64.1)	0.8683
Stable ischemic heart disease, no. (%)	8404 (94.9)	7714 (94.8)	690 (96.4)	0.0628	675 (94.7)	687 (96.3)	0.1590
Acute coronary syndrome, no. (%)	452 (5.1)	426 (5.2)	26 (3.6)	0.0628	38 (5.3)	26 (3.7)	0.1248
Previous myocardial infarction, no. (%)	2693 (30.4)	2429 (29.8)	264 (36.9)	0.0001	255 (35.8)	263 (36.9)	0.6569
History of heart failure, no. (%)	388 (4.4)	345 (4.2)	43 (6.0)	0.0354	30 (4.2)	42 (5.9)	0.1830
Left ventricular ejection fraction, %	57 ± 10 (61, 54-64)	58 ± 10 (61, 54-64)	57 ± 11 (61, 53-64)	0.3917	57 ± 11 (61, 53-64)	57 ± 11 (61, 53-64)	0.7928
Percutaneous coronary intervention, no. (%)	3682 (41.6)	3412 (41.9)	270 (37.7)	0.0296	339 (47.6)	267 (37.5)	0.0001
Coronary artery bypass grafting, no. (%)	1172 (13.2)	1022 (12.6)	150 (21.0)	<0.0001	98 (13.7)	150 (21.0)	0.0004
History of aortic dissection, no. (%)	52 (0.6)	-	52 (7.3)	-	-	52 (7.3)	-
Aortic aneurysm, no. (%)	343 (3.9)	-	343 (47.9)	-	-	342 (48.0)	-
Peripheral arterial disease, no. (%)	273 (3.1)	-	273 (38.1)	-	-	271 (38.0)	-
Other arterial diseases, no. (%)	70 (0.8)	-	70 (9.8)	-	-	70 (9.8)	-
Medications at final follow-up
Antiplatelets, no. (%)	4707 (53.2)	4322 (53.1)	385 (53.8)	0.7285	386 (54.1)	383 (53.7)	0.9154
Anticoagulants, no. (%)	1449 (16.3)	1302 (16.0)	147 (20.5)	0.0017	135 (18.9)	147 (20.6)	0.4646
Renin-angiotensin inhibitors, no. (%)	3342 (37.7)	3049 (37.5)	293 (40.9)	0.0704	294 (41.2)	291 (40.8)	0.9143
Beta-blockers, no. (%)	3826 (43.2)	3497 (43.0)	329 (46.0)	0.1250	331 (46.4)	329 (46.1)	0.9576
Calcium-channel blockers, no. (%)	3448 (38.9)	3155 (38.8)	293 (40.9)	0.2631	273 (38.3)	291 (40.8)	0.3572
Diuretics, no. (%)	1030 (11.6)	908 (11.2)	122 (17.0)	<0.0001	106 (14.9)	122 (17.1)	0.2784
Statins, no. (%)	4239 (47.9)	3923 (48.2)	316 (44.1)	0.0386	332 (46.6)	315 (44.2)	0.3947
Length of follow-up, days	1072 ± 503 (1095, 719-1469)	1074 ± 505 (1094, 720-1470)	1050 ± 484 (1098, 700-1467)	0.2317	1072 ± 493 (1099, 701-1469)	1049 ± 484 (1096, 697-1467)	0.3615

Plus-minus values are means ± standard deviation, with the median and interquartile range. CAD, coronary artery disease. Body mass index is the weight divided by the square of the height.

### Incidence of cancer development and the presence of polyvascular disease

**Figure 2.**
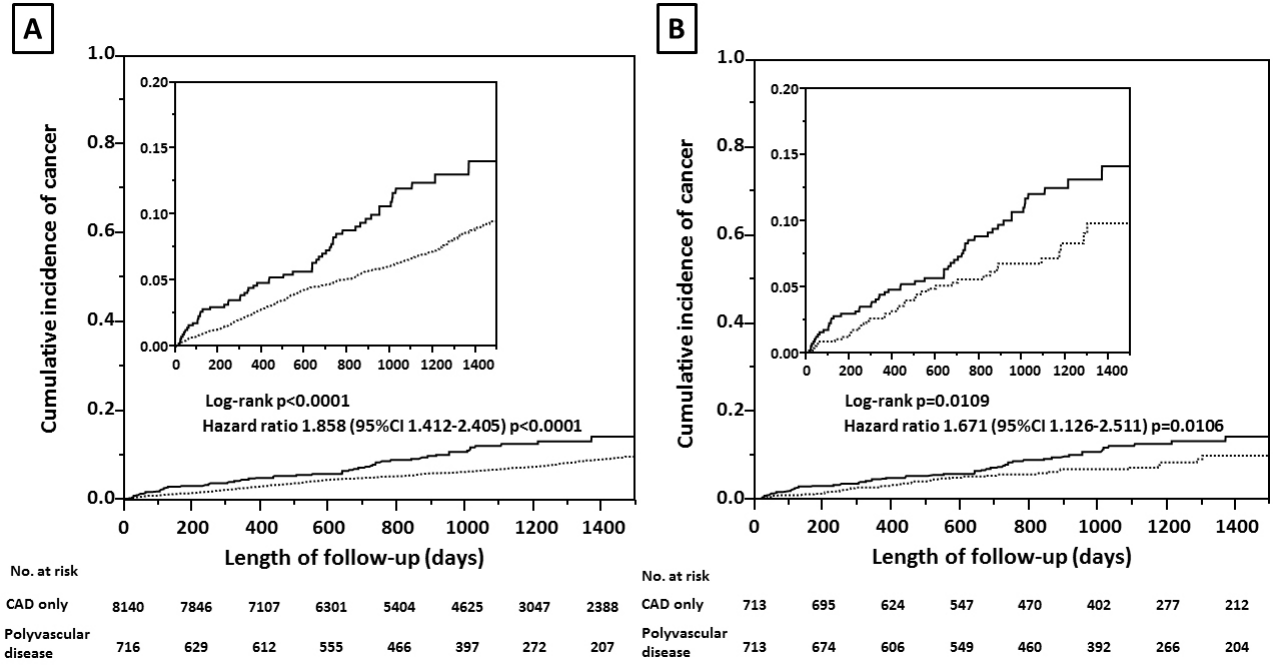
Cumulative incidence of cancer in the two cohorts with coronary artery disease (CAD) only (dotted line) and polyvascular disease (solid line) on crude analysis (A) and propensity score-matched analysis (B).

**Figure 3.**
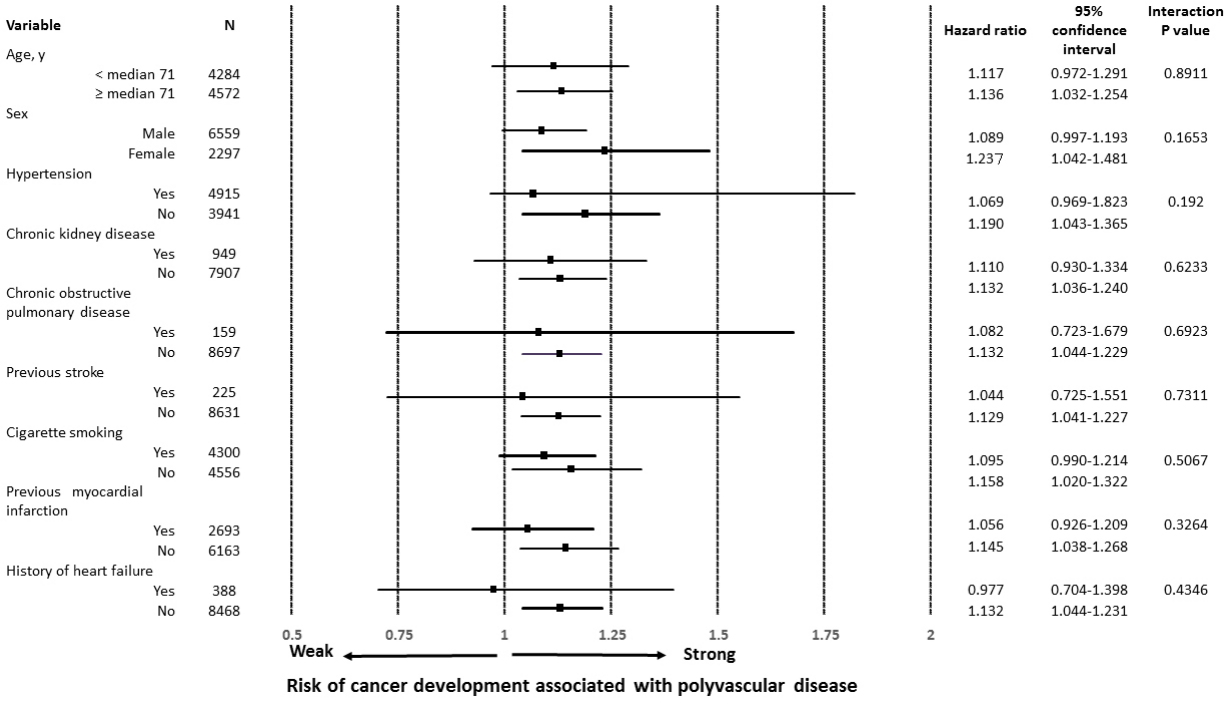
Interaction analysis of different clinical characteristics between the two cohorts based on the presence of polyvascular disease on the incidence of cancer.

A number of patients with newly diagnosed cancer was 396 in the cohort with CAD only and 63 in the cohort with polyvascular disease (4.9% vs. 8.8%, *P* < 0.0001), indicating a difference in the cumulative incidence of cancer between the two cohorts (Figure 2A). After a well-balanced propensity score matching procedure for confounding factors among the baseline characteristics, including age, male sex, hypertension, chronic kidney disease, chronic obstructive pulmonary disease, and cigarette smoking, previous myocardial infarction, and heart failure, with a C-statistic of 0.7 (Table 1), a higher incidence of cancer was still found in the cohort with polyvascular disease (Figure 2B). Even when limited to patients treated with invasive coronary therapies, a difference in the incidence of cancer was relatively unchanged between the two cohorts (4.7% vs. 8.3%, *P* = 0.0022). The distribution of organ sites of cancer development was not significantly different between the two cohorts (Table 2).On both univariate and multivariate Cox proportional hazards regression analyses adjusting for age, male sex, hypertension, chronic kidney disease, chronic obstructive pulmonary disease, and cigarette smoking, the presence of polyvascular disease was one of the principal contributors to the incidence of cancer, with a 36% higher relative risk than that of the cohort with CAD only (Table 3). Interactions of different clinical characteristics between the two cohorts were not significant for the presence of polyvascular disease on the incidence of cancer (Figure 3). A total of 548 patients (6.2% of the study patients) died during follow-up, 197 (2.2%) died due to cardiovascular disease, and 123 died (1.4%) due to cancer. All-cause mortality (15.2% vs. 5.4%, *P* < 0.0001), cardiovascular mortality (5.2% vs. 2%, *P* < 0.0001), and cancer mortality (2.5% vs. 1.3%, *P* = 0.0073) were all higher in the cohort with polyvascular disease than that in the cohort with CAD only, showing a large difference with time (Supplementary Figure S1).

**Table 2.** Organ Sites of Cancer in the Two Cohorts: with a Coronary Artery Disease (CAD) Only and with a Polyvascular Disease.

Cancer site, no. (%)	CAD only N = 396	Polyvascular disease N = 63	*P*-value
Oral cavity and pharynx	12 (3.0)	2 (3.2)	0.9507
Digestive system	183 (46.2)	28 (44.4)	0.7937
Esophagus	12 (3.0)	3 (4.8)	0.4727
Stomach	64 (16.2)	6 (9.5)	0.1734
Small intestine	4 (1.0)	0 (0)	0.4230
Colon	52 (13.1)	7 (11.1)	0.6563
Rectum	6 (1.5)	1 (1.6)	0.9654
Anus, anal canal & anorectum	0 (0)	0 (0)	1.0000
Liver and intrahepatic bile duct	22 (5.6)	8 (12.7)	0.0331
Gallbladder and biliary tract	10 (2.5)	1 (1.6)	0.6512
Pancreas	12 (3.0)	2 (3.2)	0.9507
Other digestive system	1 (0.3)	0 (0)	0.6897
Respiratory system	57 (14.4)	11 (17.5)	0.5245
Larynx	3 (0.8)	0 (0)	0.4882
Lung and bronchus	53 (13.4)	11 (17.5)	0.3856
Other respiratory organs	1 (0.3)	0 (0)	0.6897
Bones and joints	2 (0.5)	0 (0)	0.5719
Soft tissues (including heart)	0 (0)	0 (0)	1.0000
Skin	9 (2.3)	1 (1.6)	0.7292
Breast	12 (3.0)	0 (0)	0.1615
Genital system	64 (16.2)	9 (14.3)	0.7053
Uterine cervix and corpus	5 (1.3)	0 (0)	0.3698
Ovary	1 (0.3)	0 (0)	0.6897
Prostate	56 (14.1)	9 (14.3)	0.9757
Other genital organs	2 (0.5)	0 (0)	0.5719
Urinary system	37 (9.3)	7 (11.1)	0.6580
Urinary bladder	21 (5.3)	4 (6.3)	0.7340
Kidney and renal pelvis	14 (3.5)	2 (3.2)	0.8847
Ureter and other urinary organs	2 (0.5)	1 (1.6)	0.3221
Eye and orbit	0 (0)	0 (0)	1.0000
Brain and nervous system	1 (0.3)	1 (1.6)	0.1352
Endocrine system	0 (0)	0 (0)	1.0000
Lymphoma	9 (2.3)	2 (3.2)	0.6637
Myeloma	4 (1.0)	1 (1.6)	0.6818
Leukemia	6 (1.5)	0 (0)	0.3254
Other and unspecified primary sites	0 (0)	1 (1.6)	0.0121

Values are numbers (% of each cohort). CAD, coronary artery disease. Pearson, *P* = 0.3807 between the two cohorts.

**Table 3.** Cox-proportional Hazard Analysis for the Incidence of Cancer.

Variable	Univariate			Multivariate		
	Chi-squared	Hazard ratio (95% confidence interval)	*P*-value	Chi-squared	Hazard ratio (95% confidence interval)	P*-*value
Age, for each 1 year	122.355	1.055 (1.044-1.066)	<0.0001	120.687	1.057 (1.046-1.068)	<0.0001
Male	25.016	1.813 (1.423-2.343)	<0.0001	34.475	2.113 (1.630-2.770)	<0.0001
Hypertension	8.221	1.316 (1.090-1.593)	0.0041	2.301	1.159 (0.958-1.407)	0.1293
Chronic kidney disease	19.491	1.785 (1.393-2.258)	<0.0001	4.403	1.312 (1.019-1.670)	0.0359
Chronic obstructive pulmonary disease	3.969	1.806 (1.011-2.953)	0.0464	0.710	1.270 (0.708-2.085)	0.3995
Cigarette smoking	3.325	1.186 (0.987-1.425)	0.0682	0.179	1.043 (0.858-1.270)	0.0672
Polyvascular disease	17.952	1.858 (1.412-2.405)	<0.0001	4.639	1.362 (1.029-1.774)	0.0312

### Incidence of cancer development and a number of the coexisting vascular disease

**Figure 4.**
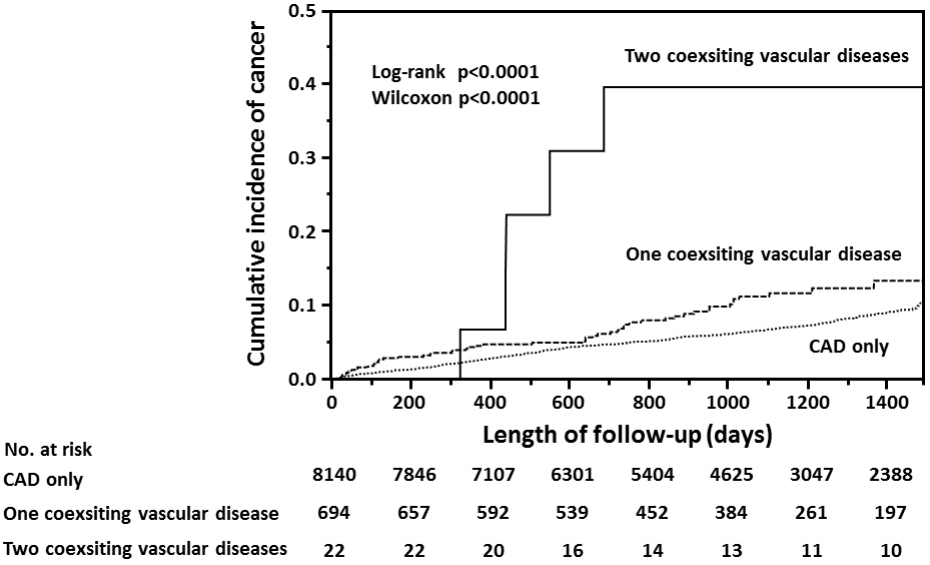
Cumulative incidence of cancer in accordance with a number of the coexisting vascular disease with coronary artery disease (CAD).

In the cohort with polyvascular disease, half of the patients had aortic disease, one-third had peripheral arterial disease, and 22 had both aortic and peripheral arterial diseases (Supplementary Table S1). By the end of follow-up, cancer was found in 28 of 373 patients (7.5%) with aortic disease, 27 of 251 (10.8%) with peripheral arterial disease, six of 22 (27.3%) with both aortic and peripheral arterial diseases, and two of 70 (2.9%) with other arterial diseases. Between the cohorts with CAD only and the two subsets of polyvascular disease (i.e., one or two coexisting vascular diseases) (Table 4), a large difference in the incidence of cancer was found in accordance with a number of the coexisting vascular disease (Figure 4).

**Table 4.** Clinical Characteristics of the Subset Cohorts of Polyvascular Disease.

Characteristic	one coexisting vascular disease N = 694	two coexisting vascular diseases N = 22	P value
Age, y	74 ± 9 (75, 69-80)	76 ± 8 (74, 72-83)	0.1739
Male, no. (%)	562 (81.0)	17 (77.3)	0.5906
Body mass index, kg/m^2^	24 ± 3 (24, 22-26)	25 ± 4 (24, 22-26)	0.1761
Hypertension, no. (%)	460 (66.3)	11 (50.0)	0.1688
Dyslipidemia, no. (%)	373 (53.8)	11 (50.0)	0.8291
Diabetes mellitus, no. (%)	206 (29.7)	4 (18.2)	0.3421
Chronic kidney disease, no. (%)	159 (22.9)	5 (22.7)	1.0000
Chronic obstructive pulmonary disease, no. (%)	30 (4.3)	2 (9.1)	0.2575
Previous stroke, no. (%)	36 (5.2)	1 (4.6)	1.0000
Cigarette smoking, no. (%)	446 (64.3)	14 (63.6)	1.0000
Stable ischemic heart disease, no. (%)	668 (96.3)	22 (100)	1.0000
Acute coronary syndrome, no. (%)	26 (3.7)	0 (0)	1.0000
Previous myocardial infarction, no. (%)	251 (36.2)	13 (59.1)	0.0411
History of heart failure, no. (%)	41 (5.9)	2 (9.1)	0.3856
Left ventricular ejection fraction, %	57 ± 11 (61, 53-64)	50 ± 14 (52, 44-62)	0.0059
Percutaneous coronary intervention, no. (%)	257 (37.0)	13 (59.1)	0.0441
Coronary artery bypass grafting, no. (%)	147 (21.2)	3 (13.6)	0.5942
History of aortic dissection, no. (%)	51 (7.4)	1 (4.6)	1.0000
Aortic aneurysm, no. (%)	322 (46.4)	21 (95.4)	<0.0001
Peripheral artery disease, no. (%)	251 (36.2)	22 (100)	<0.0001
Other arterial diseases, no. (%)	70 (10.1)	-	-
Medications at final follow-up Antiplatelets, no. (%)	372 (53.6)	13 (59.1)	0.6688
Anticoagulants, no. (%)	142 (20.5)	5 (22.7)	0.7896
Renin-angiotensin inhibitors, no. (%)	284 (40.9)	9 (40.9)	1.0000
Beta-blockers, no. (%)	322 (46.4)	7 (31.8)	0.1983
Calcium-channel blockers, no. (%)	285 (41.1)	8 (36.4)	0.8246
Diuretics, no. (%)	116 (16.7)	6 (27.3)	0.2427
Statins, no. (%)	308 (44.4)	8 (36.4)	0.5184
All-cause death, no. (%)	103 (14.8)	6 (27.3)	0.1272
Cardiovascular death, no. (%)	36 (5.2)	1 (4.6)	1.0000
Cancer death, no. (%)	16 (2.3)	2 (9.1)	0.1025
Length of follow-up, days	1046 ± 482 (1096, 697-1464)	1174 ± 549 (1199, 666-1600)	0.2223

Plus-minus values are means ± standard deviation, with the median and interquartile range. CAD, coronary artery disease. Body mass index is the weight divided by the square of the height.

## Discussion

The results showed the significantly high incidence of cancer in patients with CAD, along with the presence of polyvascular disease, as compared to that in patients with CAD only. A large difference in the incidence of cancer was also found in accordance with a number of the coexisting vascular disease with CAD. An adjustment of shared common risks between atherosclerosis and cancer demonstrated polyvascular disease as an independent contributor to the incidence of cancer. These findings imply that the presence of polyvascular disease, as a surrogate marker of the severity of atherosclerosis, would likely concern cancer development in patients with CAD.

Based on our previous study that showed a higher incidence of cancer in patients with atherosclerotic cardiovascular disease than that in those with non-atherosclerotic cardiovascular disease ^(6)^, the present study addressed the next question, whether the severity of atherosclerosis may further impact cancer development. It was assumed that the presence of polyvascular disease is an indicator of the severity of atherosclerosis in patients with CAD. Because of the heterogeneous features of atherosclerosis, which are mediated through various mechanisms, including endothelial dysfunctions, oxidized lipoproteins, inflammations, and genetic associations, et al., based on long-term unhealthy lifestyle behaviors ^(12)^, precise quantification of the severity of atherosclerosis has been limited clinically. So far, cumulative verifications of increased adverse cardiovascular events in the setting of polyvascular disease confirm that this phenotype represents the severity of atherosclerosis ^(8), (13)^. In fact, cardiovascular mortality was higher in the cohort with polyvascular disease than that in the cohort with CAD only in the present study. As one of the considerations, even though comorbidities were carefully evaluated using the SHIP surveillance system, the possibility of silent atherosclerotic cardiovascular disease could not be ruled out. Novel clinical modalities for quantitative estimates of the severity of atherosclerosis need to be developed in the near future ^(14)^.

The research field of cardio-oncology has grown because of the substantial increase in cancer survivors suffering from subsequent cardiovascular complications ^(15)^, and a possible link of cardiovascular disease with cancer development has also now come into focus ^(4), (5), (16)^. Regarding the purpose of the present study to address the potential association of the severity of atherosclerosis (i.e., the presence of polyvascular disease) with cancer development, we need to consider an influence of various shared fields to develop atherosclerosis and cancer. In the present study, nearly half of the patients had a history of cigarette smoking, which is a well-recognized risk factor for both atherosclerotic cardiovascular disease and cancer ^(17)^, and the prevalence of smokers was higher in the cohort with polyvascular disease than that in the cohort with CAD only. Nevertheless, cigarette smoking was found to be a weak hazard risk for an incidence of cancer. As one of the possibilities, enforced cessation of cigarette smoking when enrolled in the SHIP surveillance system may have affected the present findings because the basic role of modification of habitual behaviors to prevent both atherosclerotic cardiovascular disease and cancer is well-established ^(17)^. In addition, there was no interaction effect of various clinical differences as to the presence of polyvascular disease on the incidence of cancer. The high incidence of cancer in the cohort with polyvascular disease was also relatively unchanged, irrespective of the presence or absence of invasive coronary therapies. According to the annual data of the National Cancer Center between 2016 and 2018 ^(18)^, a crude annual incidence of cancer in a cumulative total number of 291,116,000 Japanese at the ages of over 15 years was 1.0%, which was clearly low, as compared with 5.2% in the present study. These also proposed a possible impact of the presence of atherosclerosis on cancer development.

Why would the severity of atherosclerosis predispose to develop cancer? Atherosclerosis is well-recognized as a chronic condition of premature vascular aging with a growth of atherosclerotic plaques, pathologically showing a loss of elasticity due to a decrease in numbers of vascular smooth muscle cells and a compensatory increase in collagen fibers ^(19)^. The phenomenon of vascular cellular senescence due to chronological aging is generally considered a natural barrier to protect against genome instability, which is the hallmark of cancer development ^(20)^, but this phenomenon has another feature, which is to secrete pro-inflammatory factors, namely, senescence-associated secretory phenotypes, to promote various aspects of cancer in a non-autonomous manner, including cancer cell proliferation, migration, invasiveness, angiogenesis, and epithelial-mesenchymal transition ^(21)^. Furthermore, with inflammation as the pivotal element in the pathogenesis of both atherosclerosis and cancer ^(22), (23), (24)^, atherogenic inflammation ^(12)^ with stress-induced premature senescence-associated secretary phenotypes ^(25), (26)^ may synergistically provide the favorable platform to develop cancer. Interestingly, in the field of clinical onco-cardiology, a combination of subtle inflammation and cancer also showed a possible risk for recurrent atherosclerotic events after percutaneous coronary intervention for the treatment of CAD ^(27)^. Recently, the presence of age-related clonal hematopoiesis, the so-called clonal hematopoiesis of indeterminate potential, has also been proposed as one of the key pro-inflammatory drivers for a link between atherosclerotic cardiovascular disease and cancer ^(28), (29)^. To investigate the central role of atherosclerosis in cancer development, we are now exploring the analysis of common extracellular vesicles between atherosclerotic cardiovascular disease and cancer ^(30)^ using practical applications of liquid biopsy ^(31), (32)^.

The present study has several limitations. First, the possibility of undetected cancer during the follow-up periods could not be excluded in the present study. However, this probability seemed to be similar between the two cohorts with CAD only and polyvascular disease; thus, it may not have affected the reliability of the present findings. Second, there were no differences in the distribution of organ sites of cancer between the two cohorts, but the present SHIP surveillance system has limited ability to identify detailed information as to each stage of cancer and also cancer therapies. We would create a more comprehensive surveillance system to analyze the characteristics of cancer in patients with any cardiovascular diseases. Third, a well-matched propensity score analysis provided a result consistent with the crude analysis regarding the association between polyvascular disease and the incidence of cancer, but this analysis could not eliminate the effects of unobserved confounding variables such as family histories, alcohol consumptions, duration of cigarette smoking, and a presence of atherosclerotic cerebrovascular disease, etc. Even though the present study was carefully constructed in accordance with the “Strengthening the Reporting of Observational Studies” in Epidemiology guidance ^(33)^, the study results need to be confirmed by nationwide studies.

### Conclusion

In the present study, an independent association of polyvascular disease with the incidence of cancer in patients with CAD was found, implying the possibility that the severity of atherosclerosis could play a pivotal role in cancer development.

## Article Information

### Conflicts of Interest

None

### Sources of Funding

This work was supported by the Sakakibara Clinical Research Grant for Promotion of Science in 2018 grant number [H-6-2018].

### Acknowledgement

The authors are sincerely grateful to Mr. Yoshikazu Yaegashi, Mr. Toshiaki Masuda, Ms. Saori Naitou, and Ms. Ikuko Shinmura for their profound contributions in assisting with this study and the data management in the Sakakibara Health Integrative Profile (SHIP) surveillance system. The authors are also very grateful to Dr. Tatsuya Murai, a distinguished cardiovascular pathologist, for his suggestion regarding the pathology of atherosclerosis and all of the staff for their clinical efforts as the heart team of the Sakakibara Heart Institute.

### Author Contributions

Makoto Suzuki: conceptualization, formal analysis, and writing of the original draft; Hitonobu Tomoike: conceptualization, data curation, supervision, methodology, writing of the review, and editing; Zhehao Dai: formal analysis, writing of the review, and editing; Toru Hosoda: writing of the review and editing; Tetsuya Sumiyoshi: project administration, writing of the review and editing; Saichi Hosoda: resources, writing of the review, and editing; and Mitsuaki Isobe: supervision, writing of the review, and editing.

### Approval by Institutional Review Board (IRB)

September 16^th^, 2003, in the Sakakibara Heart Institute (no. 11000304)

### Trial Registration

ClinicalTrials.gov. number: NCT04198896.

### Disclaimer

Mitsuaki Isobe is one of the Associate Editors of JMA Journal and on the journal’s Editorial Staff. He was not involved in the editorial evaluation or decision to accept this article for publication at all.

## Supplement

Supplementary Table S1

Supplementary Figure S1
