# Supplementary material for: Polyvascular Disease and the Incidence of Cancer in Patients with Coronary Artery Disease
Source: JMA J. 2022 Sep 26;5(4):498–509. doi: 10.31662/jmaj.2022-0098 (PMC9646297; doi:10.31662/jmaj.2022-0098)
Supplement: Supplementary file 1 — Supplementary Table S1 [file 2433-3298-5-4-0498-s001.pdf]

Table S1. Clinical characteristics of patients with each vascular disease in the cohort with polyvascular disease

Characteristic	Aortic disease N=373	Peripheral arterial disease N=251	Both aortic and peripheral arterial diseases N=22	Other arterial diseases N=70	P value
Age, y	74 ± 9 (75, 70-80)	74 ± 8 (75, 69-79)	76 ± 8 (74, 72-83)	72 ± 11 (74, 67-79)	0.0593
Male, no. (%)	309 (82.8)	207 (82.5)	17 (77.3)	46 (65.7)	0.0076
Body mass index, kg/m^2	24 ± 8 (24, 22-26)	25 ± 9 (24, 22-26)	25 ± 4 (24, 22-26)	25 ± 7 (24, 22-26)	0.9670

Hypertension, no. (%)	255 (68.4)	163 (64.9)	11 (50.0)	42 (60.0)	0.1986
Dyslipidemia, no. (%)	199 (53.4)	135 (53.8)	11 (50.0)	39 (55.7)	0.9686
Diabetes mellitus, no. (%)	65 (17.4)	115 (45.8)	4 (18.2)	26 (37.1)	<0.0001
Chronic kidney disease, no. (%)	80 (21.5)	66 (26.3)	5 (22.7)	13 (18.6)	0.4191
Chronic obstructive pulmonary disease, no. (%)	17 (4.6)	9 (3.6)	2 (9.1)	4 (5.7)	0.6105
Previous stroke, no. (%)	14 (3.8)	17 (6.8)	1 (4.6)	5 (7.1)	0.3317
Cigarette smoking, no. (%)	241 (64.6)	180 (71.7)	14 (63.6)	25 (35.7)	<0.0001
Stable ischemic heart disease, no. (%)	358 (96.0)	244 (97.2)	22 (100)	66 (94.3)	0.4996
Acute coronary syndrome, no. (%)	15 (4.0)	7 (2.8)	0 (0)	4 (5.7)	0.4996
Previous myocardial infarction, no. (%)	139 (37.3)	94 (37.5)	13 (59.1)	18 (25.7)	0.0372
History of heart failure, no. (%)	17 (4.6)	21 (8.4)	2 (9.1)	3 (4.3)	0.1860

Left ventricular ejection fraction, %	58 ± 10	58 ± 9	50 ± 14	55 ± 12	0.0217
	(62, 55-64)	(61, 55-64)	(52, 44-62)	(59, 50-64)	
Percutaneous coronary intervention, no. (%)	135 (36.2)	100 (39.8)	13 (59.1)	22 (31.4)	0.0976
Coronary artery bypass grafting, no. (%)	74 (19.8)	62 (24.7)	3 (13.6)	11 (15.7)	0.2328
Medications at final follow-up					
Antiplatelets, no. (%)	184 (49.3)	144 (57.4)	13 (59.1)	44 (62.9)	0.0771
Anticoagulants, no. (%)	75 (20.1)	52 (20.7)	5 (22.7)	15 (21.4)	0.9858
Renin-angiotensin inhibitors, no. (%)	148 (39.7)	106 (42.2)	9 (40.9)	30 (42.9)	0.9134
Beta-blockers, no. (%)	173 (46.4)	115 (45.8)	7 (31.8)	34 (48.6)	0.5740
Calcium-channel blockers, no. (%)	156 (41.8)	93 (37.1)	8 (36.4)	36 (51.4)	0.1670
Diuretics, no. (%)	55 (14.8)	49 (19.5)	6 (27.3)	12 (17.1)	0.2495

Statins, no. (%)	166 (44.5)	103 (41.0)	8 (36.4)	39 (55.7)	0.1483
All-cause death, no. (%)	52 (13.9)	44 (17.5)	6 (27.3)	7 (10.0)	0.1408
Cardiovascular death, no. (%)	22 (5.9)	13 (5.2)	1 (4.6)	1 (1.4)	0.4898
Cancer death, no. (%)	5 (1.3)	10 (4.0)	2 (9.1)	1 (1.4)	0.0363
Length of follow-up, days	1064 ± 480 (1110, 728-1470)	1008 ± 473 (1047, 673-1450)	1174 ± 549 (1199, 666-1600)	1091 ± 520 (1114, 688-1483)	0.2438

Plus-minus values are means ± standard deviation with the median and interquartile range. Body mass index is the weight divided by the square of the height.
