# Supplementary figures and images for: Polyvascular Disease and the Incidence of Cancer in Patients with Coronary Artery Disease
Source: JMA J. 2022 Sep 26;5(4):498–509. doi: 10.31662/jmaj.2022-0098 (PMC9646297; doi:10.31662/jmaj.2022-0098)

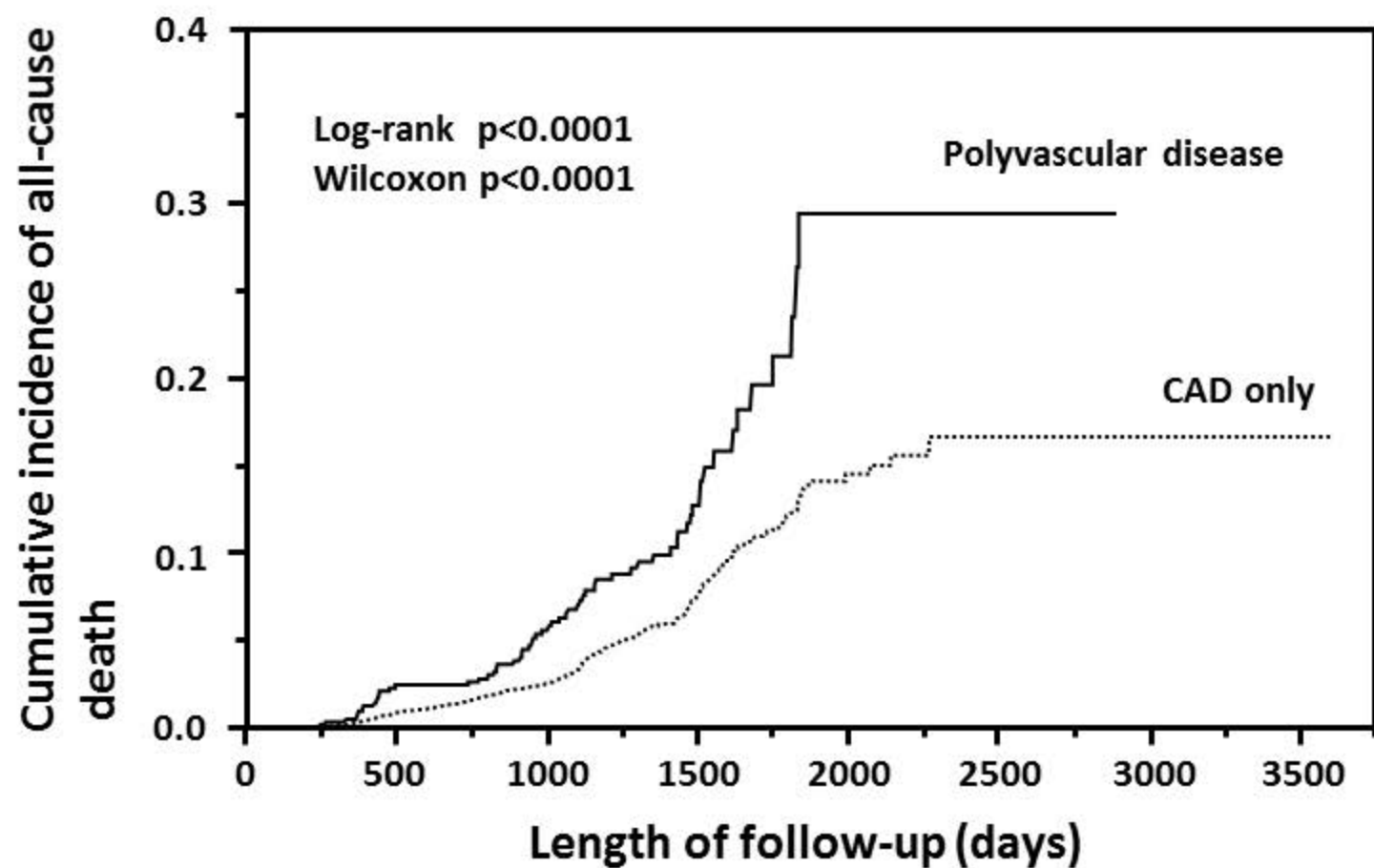

No. at risk

CAD only

Polyvascular disease

|      |      |      |      |     |    |    |   |
|------|------|------|------|-----|----|----|---|
| 8140 | 6651 | 4784 | 1623 | 217 | 32 | 15 | 3 |
| 716  | 578  | 414  | 135  | 10  | 3  | -  | - |

Supplement: Supplementary file 2 — Supplementary Figure S1 [file 2433-3298-5-4-0498-s002.pdf]
